# Association Between *rs920778* Polymorphisms and Cancer Risk: An Updated Meta-Analysis

**DOI:** 10.1155/genr/2340176

**Published:** 2025-07-03

**Authors:** Lihua Xu, Jiang Deng, Lili Gong, Yajuan Chen, Gang Hu

**Affiliations:** ^1^Wuhan Children's Hospital (Wuhan Maternal and Child Healthcare Hospital), Tongji Medical College, Huazhong University of Science & Technology, Wuhan 430016, Hubei, China; ^2^Hubei No. 3 People's Hospital of Jianghan University, Wuhan, China

**Keywords:** cancer, genetic susceptibility, HOTAIR rs920778 polymorphism

## Abstract

**Background:** A growing number of studies are exploring the association between HOTAIR rs920778 polymorphisms and cancer risk, but to date, there has been controversy and uncertainty. Preliminary evidence suggests that this polymorphism may influence cancer susceptibility, particularly in Asian populations and specific cancer types such as cervical cancer (CC) and breast cancer (BC). We therefore conducted an updated meta-analysis to accurately assess the association of the HOTAIR rs920778 polymorphism with cancer risk.

**Method:** Comprehensive literature searches were performed in PubMed, Embase, and Web of Science up to September 8, 2023. Inclusion criteria included case-control studies with allele frequency data for both cases and controls. A total of 29 case-control studies were selected for quantitative analysis. Crude odds ratios (ORs) and 95% confidence intervals (CIs) were calculated using Stata software (Version 11) to evaluate the association between the rs920778 polymorphism and cancer risk. Heterogeneity and publication bias were assessed using chi-square tests, *I*^2^ statistics, and funnel plots with Egger's test.

**Results:** Our analysis of the results found a significant association between the rs920778 polymorphism and cancer susceptibility. In Asian populations, all five genetic models of the rs920778 polymorphism have been shown to increase overall cancer susceptibility. At the same time, we performed stratified analyses based on cancer type and found that all genetic models revealed significantly increased susceptibility to CC in Asian populations. Conversely, the heterozygote model of rs920778 demonstrated significantly reduced susceptibility to BC, with consistent effects across racial groups.

**Conclusions:** Our meta-analysis demonstrated that the HOTAIR rs920778 polymorphism may be a risk factor for cancer but may serve as a protective factor for BC. Future studies require larger sample sizes and gene function analysis, suggesting that the rs920778 polymorphism could serve as a genetic biomarker to guide targeted therapies or cancer screening.

## 1. Introduction

Recently, long noncoding RNAs (lncRNAs) have attracted the attention of many researchers due to their comprehensive functions. lncRNAs are endogenous RNAs larger than 200 nucleotides that do not encode any proteins [1]. In humans, > 10,000 lncRNAs have been identified that participate in cell growth, proliferation, survival, metabolism, differentiation, development, and many disease biological processes through genomic packaging, genomic imprinting, gene-regulated alternative splicing, chromatin organization, dose compensation, etc. [2]. Many studies have shown that lncRNAs may affect cancer-related genes by regulating transcription and other biological processes and play a key role in tumor occurrence and development [3]. The lncRNA HOX transcribed antisense RNA (HOTAIR) is one of these RNAs, and the HOTAIR gene is located on the long arm of chromosome 12 (12q13.13). As an oncogene, HOTAIR is expressed in a variety of human cancers, and its overexpression is associated with the proliferation, invasion, progression, and metastasis of cancer cells, as well as a low survival rate [4]. HOTAIR single-nucleotide polymorphisms (SNPs) have been studied as potential cancer susceptibility sites and have been associated with an increased risk of human cancers, such as breast cancer (BC) [5–7], esophageal squamous cell carcinoma [1], stomach cancer [8], lung cancer [9], and colorectal cancer [10]. This suggests that HOTAIR may be an oncogene in different types of cancer. Many studies have shown that overexpression of HOTAIR is a risk factor for poor tumor prognosis during tumor invasion and progression [11]. rs920778, located in intron 2 of the HOTAIR gene, is a new intron enhancer. The rs920778 polymorphism has a genotype-specific effect on HOTAIR expression, resulting in higher HOTAIR expression in T allele carriers, which affects susceptibility to cancer [12]. However, studies have also shown that the rs920778 polymorphism is not associated with other types of cancer, except for lung cancer, and that the rs920778 polymorphism does not affect the total presence of cancer [13]. To date, SNPs in HOTAIR have been extensively studied in malignant tumors. Studies on the strength of the association between the rs920778 polymorphism and cancer risk have been inconsistent. Therefore, we conducted this meta-analysis to provide a more comprehensive meta-analysis of the relationship between the rs920778 polymorphism and cancer based on the synthesis of current relevant studies. We collected all published case and control studies using various research methods and models to detail the role of the HOTAIR rs920778 gene up to September 2023.

## 2. Methods and Materials

### 2.1. Publication Search

Comprehensive literature searches were conducted in PubMed, Embase, and Web of Science up to September 8, 2023. The search terms included the following: “Polymorphism or SNP or mutation or variation or allele or genotype,” “HOTAIR or HOX antisense intergenic RNA,” “rs920778,” and “cancer or tumor or malignancy or neoplasm.” In addition, manual searches of reference lists from eligible publications were performed by two authors (LHX and GH) to identify additional relevant studies.

### 2.2. Inclusion and Exclusion Criteria

#### 2.2.1. Inclusion Criteria

Inclusion criteria included (1) case-control studies, (2) studies focusing on the relationship between HOTAIR rs920778 polymorphism and cancer risk, and (3) studies containing detailed allele frequencies in both cases and controls.

#### 2.2.2. Exclusion Criteria

The exclusion criteria excluded the following: (1) reviews, editorials, or studies without primary data and (2) studies lacking sufficient genotype data for analysis.

### 2.3. Data Extraction and Quality Assessment

Inclusion criteria were case-control studies published up to September 2023, and articles should include at least allele frequencies in both cases and controls. The following data were extracted from each study: basic article information (first author, year of publication, region, ethnicity, control source, sample size, allele and genotype frequency, age and sex matching status, and cancer type). Discrepancies in data extraction were resolved through discussion with a third author (JD).

### 2.4. Statistical Analysis

Hardy–Weinberg equilibrium (HWE) was assessed for all eligible studies using chi-square tests. Odds ratios (ORs) with 95% confidence intervals (CIs) were calculated to evaluate the association between HOTAIR rs920778 polymorphism and cancer risk. Five genetic models were analyzed as follows: allele model (T vs. C), dominant model (CT + TT vs. cervical cancer [CC]), recessive model (TT vs. CC + CT), homozygous model (TT vs. CC), and heterozygous model (TC vs. CC). Heterogeneity was assessed using chi-square tests and *I*^2^ statistics. A random-effects model was applied if significant heterogeneity was detected (*p* < 0.05 or *I*^2^ > 50%); otherwise, a fixed-effect model was used. Subgroup analyses were performed based on ethnicity, cancer type, and other covariates. Publication bias was evaluated using funnel plots and Egger's test.

All statistical analyses were performed using STATA software 11.0 (StataCorp LP, College Station, TX). A *p* value < 0.05 was considered statistically significant.

## 3. Results

### 3.1. Characteristics of Eligible Studies

**Figure 1:**
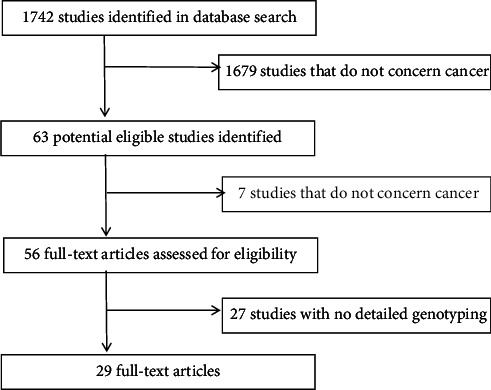
Study flowchart explaining the selection of the 30 eligible articles.

The study selection process is illustrated in Figure 1. A total of 63 publications were initially identified, of which 29 studies [1, 5–7, 12, 14–35], comprising 9433 cases and 12,682 controls, were included in the meta-analysis. Among these, 22 studies focused on Asian populations, and seven on Caucasians in Table 1. The HWE test results indicated that 24 of the 29 studies were in equilibrium (*p* > 0.05).

### 3.2. Quantitative Synthesis

**Figure 2:**
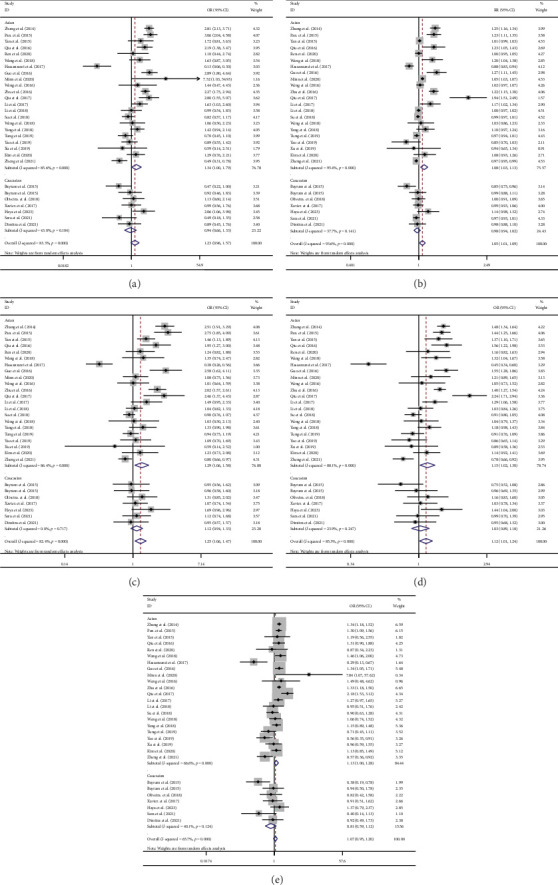
Forest plots of HOTAIR *rs920778* polymorphism and cancer for all eligible studies. (a) Homozygote model: TT versus CC. (b) Dominant model: CT + TT versus CC. (c) Recessive model: TT versus TC + CC. (d) Additive model: T versus C. (e) Heterozygote model: TC versus CC.

This study examined the association of the HOTAIR rs920778 C > T polymorphism with cancer risk. A total of five genetic models of the rs920778 polymorphism were tested for OR and 95% CIs. The main meta-analysis results are shown in Figure 2. Overall analysis revealed that for the population as a whole, mutations in this gene are associated with an increased risk of cancer in dominant, invisible, and additive models (CT + TT vs. CC: OR = 1.051, 95% CI: 1.011–1.092; TT vs. CC + CT: OR = 1.251, 95% CI: 1.063–1.472; T vs. C: OR = 1.122, 95% CI: 1.012–1.244). Ethnic stratification analysis showed that in homozygous, dominant, recessive, and additive models, this gene mutation showed an increased risk of cancer in Asian populations (TT vs. CC: OR = 1.337, 95% CI: 1.001–1.786; CT + TT vs. CC: OR = 1.076, 95% CI: 1.026–1.128; TT vs. CC + CT: OR = 1.293, 95% CI: 1.058–1.580; T vs. C: OR = 1.150, 95% CI: 1.021–1.295). Stratified tumor analysis showed that the mutation of this gene significantly increased the risk of CC based on all five gene models (TT vs. CC: OR = 1.7, *I*^2^ < 50%; CT + TT vs. CC: OR = 1.294, 95% CI: 1.112–1.505, *p*=0.281, *I*^2^ < 25%; CT + TT vs. CC: OR = 1.469, 95% CI: 1.073–2.012, *p*=0.045, *I*^2^ < 75%; T vs. C: OR = 1.250, 95% CI: 1.067–1.465, *p*=0.028, *I*^2^ < 75%; TC vs. CC: OR = 1.208, 95% CI: 1.054–1.386, *p*=0.830, *I*^2^ < 25%). In the heterozygous model, mutation of this gene significantly reduced the risk of BC (TC vs. CC: OR = 0.534, 95% CI: 0.390–0.731, *p* < 0.05). In the dominant model, this gene mutation was associated with a decreased risk of BC (CT + TT vs. CC: OR = 0.511, 95% CI: 0.254–1.026). In the additive model, this gene mutation significantly increased the risk of cancer in LC (T vs. C: OR = 1.156, 95% CI: 1.018–1.313, *p*=0.462, *I*^2^ < 25%). In homozygous models, mutations in this gene tended to increase the risk of LC (TT vs. CC: OR = 1.225, 95% CI: 0.956–1.571). In recessive and dominant models, this gene mutation was associated with an increased risk of OC (CT + TT vs. CC: OR = 1.195, 95% CI: 1.007–1.418; TT vs. CC + CT: OR = 1.353, 95% CI: 1.051–1.5744).

### 3.3. Publication Bias and Sensitivity Analysis

**Figure 3:**
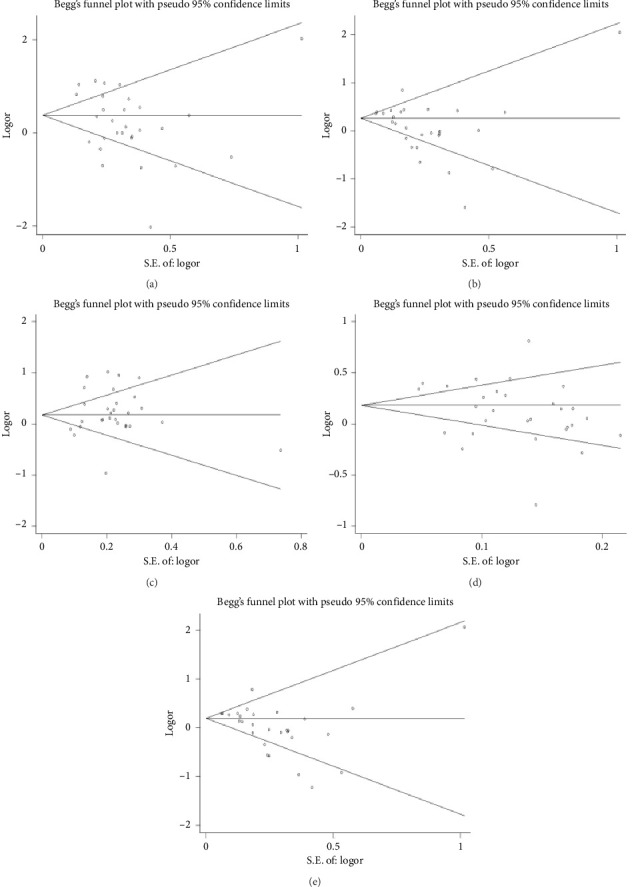
Funnel plots for all publications. (a) Homozygote model. (b) Dominant model. (c) Recessive model. (d) Additive model. (e) Heterozygote model.

No significant publication bias was detected using Egger's test and funnel plots (Figure 3). Sensitivity analyses confirmed the robustness of the results.

## 4. Discussion

Cancer continues to be one of the major public health threats worldwide [37]. By 2022, 1,918,030 new cancer cases and 609,360 cancer deaths were estimated to occur in the United States, making cancer the second leading cause of death in the United States and the leading cause of death worldwide [38]. Many factors have been found to contribute to the development and progression of cancer, such as high BMI, infections, smoking, and heavy alcohol consumption. In addition to the above factors, genetic variation has received increasing attention as one of the risk factors for cancer [36]. Among genetic risk factors, SNPs in some genetically modified oncogenes or tumor-suppressor genes are involved in the onset and progression of the disease [39]. HOTAIR is a well-studied lncRNA, and several polymorphisms of the HOTAIR gene have been found, which may affect its transcriptional activity. Some of these polymorphisms are thought to be genetic susceptibility factors for cancer [40]. Zhang et al. determined that the HOTAIR SNP rs920788 affects its specific expression through intron enhancers [41]. Zhang et al. first studied the relationship between HOTAIR gene polymorphisms and cancer susceptibility and reported that HOTAIR rs920778 was significantly associated with the risk of esophageal squamous cell carcinoma in the Chinese population [1]. In 2020, Liu et al. found that the HOTAIR rs920778 C > T polymorphism was significantly associated with overall cancer risk and gastrointestinal cancer risk in subgroup analysis [36]. In 2017, Yating Ge et al.'s analysis found a significant association between the rs920778 polymorphism and increased cancer susceptibility in both homozygous and recessive models. Stratified analysis was also performed according to cancer type, and a significant increase in susceptibility to esophageal squamous cell carcinoma was found in all genetic models and a significant increase in susceptibility to gastric cancer in the dominant model [40]. However, in 2016, Tian et al. did not find a significant association between the HOTAIR rs920778 polymorphism and cancer risk [41]. There are an increasing number of studies on the HOTAIR rs920788 polymorphism, and results of these studies in regard to susceptibility to different cancer types in different populations are inconsistent. We therefore pooled 29 studies to assess the association of HOTAIR rs920778 polymorphisms with cancer susceptibility.

Our meta-analysis produced results consistent with previous studies, as well as inconsistent results. For example, Ge et al. [40], Bayram et al. [5], Pan et al. [14], and Zhang et al. [42] all showed a significant association between the rs920778 polymorphism and cancer susceptibility, which was consistent with the results of our meta-analysis. Zhang et al. reported that the rs920778 polymorphism was associated with cancer susceptibility in Asian populations but not in Turkish populations [42]. Our meta-analysis showed that homozygous (TT vs. CC: OR = 1.337, 95% CI: 1.001–1.786), dominant (CT + TT vs. CC: OR = 1.076, 95% CI: 1.026–1.1280), recessive (TT vs. TC + CC: OR = 1.293, 95% CI: 1.058–1.580), and additive (T vs. C: under the OR = 1.150, 95% CI: 1.021–1.295) genetic models and HOTAIR rs920778 gene mutation revealed an increased risk of cancer in Asian populations but no significant association in Caucasian populations. This may be related to the differences in HOTAIR allele frequencies between Asians and other ethnicities. Different races have different lifestyles and are affected by different environmental factors. It is suggested that the rs920778 polymorphism is associated with cancer susceptibility in the Asian population. In 2023, Wang et al.'s study showed that the HOTAIR rs920778 polymorphism had no significant relationship with BC risk, and the rs920778 polymorphism showed a completely opposite association with BC risk in West Asian and East Asian populations in ethnic subgroup analysis [43]. Yan et al. indicated that the rs920778 polymorphism may be a risk factor for BC [6]. This suggests that the association between the rs920778 polymorphism and BC risk is currently inconsistent. In the current meta-analysis, analysis of a heterozygous genetic model based on the rs920778 locus showed that mutations in this gene were significantly negatively associated with BC risk (OR = 0.534, 95% CI: 0.390-0.731, *p* < 0.05). This clarifies the relationship between the rs920778 polymorphism and BC. In 2018, Li et al. found that the expression of rs920778 was not clearly associated with the risk of BC, CC, and OC [2]. In 2016, Qiu et al. reported for the first time that the TT genotype of HOTAIR rs920778 significantly increased the risk of neutron CC in the Chinese population [31]. The stratified analysis of tumor types in our meta-analysis showed that under 5 genetic models (OR = 1.733, 95% CI: 1.242–2.417, *p*=0.106, *I*^2^ < 50%; OR = 1.294, 95% CI: 1.112–1.505, *p*=0.281, *I*^2^ < 25%; OR = 1.469, 95% CI: 1.073–2.012, *p*=0.045, *I*^2^ < 75%; OR = 1.250, 95% CI: 1.067–1.465, *p*=0.028, *I*^2^ < 75%; OR = 1.208, 95% CI: 1.054–1.386, *p*=0.830, *I*^2^ < 25%), HOTAIR rs920778 gene mutation significantly increased the risk of CC in Asians, which confirms that the rs920778 polymorphism increases the risk of CC in Asian ethnic groups and suggests that it may serve as a prognostic marker for CC.

## 5. Conclusion

The HOTAIR rs920778 polymorphism is an important cancer risk factor that significantly influences susceptibility to CC and BC. The rs920778 polymorphism may affect the expression of HOTAIR, thereby altering cancer-related pathways. For BC, the protective effect may be related to different gene regulations or interactions with hormonal factors. HOTAIR is involved in the modification and regulation of epigenesis in the cells via targeting chromatin-modifying complex occupancy/localization. Cancer is a multifactorial malignant disease that likely arises from complex interactions between genetic mutations, environmental changes, lifestyle, diet, age, and sex. In our meta-analysis, we only focused on the HOTAIR polymorphisms, while the fundamental underlying mechanisms cannot be explained citing [17, 23]. Our meta-analysis confirmed the association between the HOTAIR rs920778 polymorphism and cancer susceptibility, especially in Asian populations, and the findings are more plausible than previous analyses. This provides accurate evidence for future cancer-related studies. However, there are some limitations to this study. For example, the small sample size for the subgroup analysis. The predominantly Asian population limits generalizability. There may be unmeasured confounders in the included studies. Future research should focus on larger, multiethnic cohorts and functional studies to elucidate the underlying mechanisms.

## Figures and Tables

**Table 1:** Main characteristics of all studies eligible for the meta-analysis.

Studies	Year	Ethnicity	Cancer type	Case			Control			*p* value (HWE)
				CC	CT	TT	CC	CT	TT	
Zhang et al. [1]	2014	Asian	Esophageal cancer	1091	826	181	1323	749	78	0.025
Bayram et al. [7]	2015	Caucasian	Breast cancer	31	52	40	15	66	41	0.14
Pan et al. [14]	2015	Asian	Gastric cancer	420	321	59	980	575	45	0
Bayram et al. [5]	2015	Caucasian	Gastric cancer	20	52	32	38	105	66	0.738
Yan et al. [6]	2015	Asian	Breast cancer	12	151	339	18	190	296	0.06
Qiu et al. [31]	2016	Asian	Cervical cancer	90	78	47	226	150	54	0
Ren et al. [21]	2020	Asian	Lung cancer	10	60	114	10	69	104	0.742
Wang et al. [18]	2018	Asian	Lung cancer	110	132	20	233	192	26	0.095
Hassanzarei et al. [17]	2017	Asian	Breast cancer	33	119	68	8	98	125	0.032
Oliveira et al. [19]	2018	Caucasian	Prostate cancer	23	50	78	27	72	81	0.105
Guo et al. [15]	2016	Asian	Cervical cancer	269	189	52	448	235	30	0.907
Minn et al. [13]	2020	Asian	Lung cancer	1	53	75	70	473	698	0.384
Weng et al. [20]	2016	Asian	Cervical cancer	4	42	50	19	134	165	0.226
Zhu et al. [16]	2016	Asian	Thyroid cancer	1257	960	183	1465	841	94	0.05
Qiu et al. [12]	2017	Asian	Ovarian cancer	235	69	25	580	78	22	8.374
Li et al. [25]	2017	Asian	Hepatocellular cancer	248	186	48	304	180	36	0.191
Xavier-Magalhães et al. [30]	2017	Caucasian	Glioma	38	116	136	25	84	90	0.438
Li et al. [33]	2018	Asian	Lung cancer	22	192	324	22	203	326	0.164
Su et al. [27]	2018	Asian	Oral squamous cell cancer	64	403	440	73	513	614	0.011
Weng et al. [20]	2018	Asian	Cervical cancer	107	92	13	165	134	19	0.226
Yang et al. [28]	2018	Asian	Neuroblastoma	191	158	44	430	310	70	0.189
Tung et al. [29]	2019	Asian	Urothelial cell carcinoma	38	176	217	55	360	447	0.119
Yao et al. [35]	2019	Asian	Gastric cancer	80	32	40	135	96	76	8.09
Xu et al. [36]	2019	Asian	Colorectal cancer	156	41	3	153	42	5	0.311
Kim et al. [26]	2020	Asian	Colorectal cancer	258	180	36	241	149	26	0.645
Zheng et al. [23]	2021	Asian	Breast cancer	53	274	498	31	281	593	0.746
Haya et al. [24]	2023	Caucasian	Colorectal cancer	35	70	39	48	70	26	0.957
Sara et al. [22]	2021	Caucasian	Lymphoma	9	49	98	7	96	156	0.082
Dimitra et al. [32]	2021	Caucasian	Cholangiocarcinoma	24	61	37	30	83	52	0.755

## Data Availability

The data supporting the findings of this study are available within the paper and its Supporting Information. Additional raw data are available from the corresponding author upon reasonable request.
