# Corrigendum to “Association Between *rs920778* Polymorphisms and Cancer Risk: An Updated Meta-Analysis”

**DOI:** 10.1155/genr/9784318

**Published:** 2025-09-18

L. Xu, J. Deng, L. Gong, Y. Chen, and G. Hu, “Association Between *rs920778* Polymorphisms and Cancer Risk: An Updated Meta-Analysis,” *Genetics Research* 2025 (2025): 2340176, https://doi.org/10.1155/genr/2340176.

In the article titled “Association Between *rs920778* Polymorphisms and Cancer Risk: An Updated Meta-Analysis”, there was an error in the article type, where the article type was incorrectly indicated as “Review Article.” The correct article type is “Research Article.”

We apologize for this error.

